# An observational study of pleiotropy and penetrance of amyotrophic lateral sclerosis associated with CAG-repeat expansion of *ATXN2*

**DOI:** 10.1038/s41431-025-01811-2

**Published:** 2025-02-16

**Authors:** Koen C. Demaegd, Aoife Kernan, Johnathan Cooper-Knock, Joke J. F. A. van Vugt, Calum Harvey, Tobias Moll, David O’Brien, Sarah Gornall, Luke Drury, Sali M. K. Farhan, Patrick A. Dion, Guy A. Rouleau, Andrea Western, Paul J. Parsons, Benjamin Mclean, Michael Benatar, Leonard H. van den Berg, Philip Van Damme, Jan Willem Dankbaar, Jeroen Hendrikse, Wouter Koole, Charlotte de Bie, Esther Hobson, Jan H. Veldink, Bart van de Warrenburg, R. Jeroen Pasterkamp, Wouter van Rheenen, Janine Kirby, Pamela J. Shaw, Michael. A. van Es

**Affiliations:** 1 Department of Neurology, Utrecht University, Utrecht, The Netherlands; 2 Sheffield Institute for Translational Neuroscience, University of Sheffield, Sheffield, UK; 3 Department of Radiology and Nuclear Medicine, Utrecht University, Utrecht, The Netherlands; 4 Department of Genetics, Utrecht University, Utrecht, The Netherlands; 5 PreventionGenetics, Part of Exact Sciences, Marshfield, WI USA; 6 Montreal Neurological Institute-Hospital, McGill University, Montreal, QC Canada; 7 Sheffield Children’s NHS Foundation Trust, Sheffield, UK; 8 University of North Carolina, Chapel Hill, NC USA; 9 University of Miami, Miller School of Medicine, Coral Gables, FL USA; 10 Neurology Department, University of Leuven (KU Leuven), Leuven, Belgium; 11 Sheffield Teaching Hospitals NHS Foundation Trust, Sheffield, UK; 12 NIHR Sheffield Biomedical Research Centre, Sheffield, UK; 13 Department of Neurology, Radboud University, Nijmegen, the Netherlands; 14 Department of Translational Neuroscience, Utrecht University, Utrecht, The Netherlands; 15 Neuroscience Institute, University of Sheffield, Sheffield, UK

**Keywords:** Disease genetics, Genetic association study

## Abstract

Spinocerebellar ataxia type 2 (SCA2) and amyotrophic lateral sclerosis (ALS) are both associated with a CAG-repeat expansion in *ATXN2* and with TDP-43-positive neuronal cytoplasmic inclusions. The two disorders have been viewed as distinct entities, where an intermediate length expansion of 31-33 CAG-repeats is associated with sporadic ALS and a full length expansion of ≥34 CAG-repeats is associated with SCA2. We report the clinical phenotype of *ATXN2*-positive patients and their relatives, identified in three specialist ALS clinics, which force a reconsideration of this dichotomy. We also report the frequency of *ATXN2* expansions in two large cohorts of ALS patients and in a population-matched cohort of controls. We report ten cases of *familial* ALS in which disease is associated with either an intermediate or a full-length *ATXN2* CAG-repeat expansion. Pedigrees and patients feature additional phenotypes including parkinsonism, dementia and essential tremor (ET). We conclude that CAG-repeat expansions in *ATXN2* exhibit pleiotropy and are associated with a disease spectrum that includes ALS, SCA2, and parkinsonism; to recognise this complexity we propose the new term ‘*ATXN2*-related neurodegeneration’. We also observed sporadic ALS associated with full-length expansions. We conclude that *ATXN2* CAG-repeat expansions, irrespective of length, should be considered a risk factor for ALS. Interrupted CAG-repeats were associated with an ALS phenotype in our data but we also identified ALS cases with uninterrupted expansions. Our findings have relevance for researchers, patients and families linked to CAG-repeat expansions in *ATXN2*.

## Background

Spinocerebellar ataxia type 2 (SCA2) and amyotrophic lateral sclerosis (ALS) are both associated with a CAG-repeat expansion in *ATXN2* and with the development of TDP-43-positive neuronal cytoplasmic inclusions [1]. ALS is particularly associated with intermediate-length expansions, with the highest risk for 31-33 CAG-repeats [2–4], and SCA2 with ≥34 CAG-repeats [5]. CAG-repeat lengths of 29 and 30 are more frequent in ALS compared to the background population but, in the largest meta-analysis including 6151 sporadic ALS patients and 7505 controls, this difference was not statistically significant [2]. Notably, ALS associated with 31-33 repeats is typically sporadic, and expansions of a similar length are found in a small but significant proportion of healthy controls [2, 6] and therefore intermediate-length *ATXN2* expansions have been interpreted as a risk factor for ALS with low penetrance. There are a small number of reports where full-length ≥34 CAG-repeat *ATXN2* expansions are associated with *familial* ALS, or the related disorder frontotemporal dementia (FTD) [7, 8], but the current consensus is that *ATXN2* CAG-repeat expansions are *not* routinely associated with familial ALS, and full-length expansions are *not* routinely associated with ALS in general [9].

Here we report ten familial ALS pedigrees associated with both intermediate and full-length *ATXN2* expansions, as well as fifteen cases of sporadic ALS associated with full-length *ATXN2* expansions. The pedigrees and patients we describe manifest ALS together with parkinsonism, and even essential tremor (ET). We propose the new term ‘*ATXN2*-related neurodegeneration’ to capture these complexities. Our observations of familial ALS associated with *ATXN2* CAG-repeat expansions cause us to conclude that penetrance may be higher than previously thought, at least within specific pedigrees. Finally, we present two separate cohort studies where we identified an intermediate or full-length *ATXN2* expansion in 2.2% of all ALS patients across both cohorts; the association was particularly marked for full-length expansions (OR = 6.5). We conclude that the phenomena we describe are not uncommon and should inform routine clinical practice for clinical geneticists and neurologists working with ALS patients.

## Results

Here we describe pedigrees and patients associated with both intermediate and full-length *ATXN2* expansions. In all cases except the Montreal patient, the most frequent ALS-associated mutation in *C9ORF72* [10] was excluded. For other cases the details of additional genetic testing are included in Supplementary Table 1.

### Full length expansions

#### Utrecht A

**Fig. 1:**
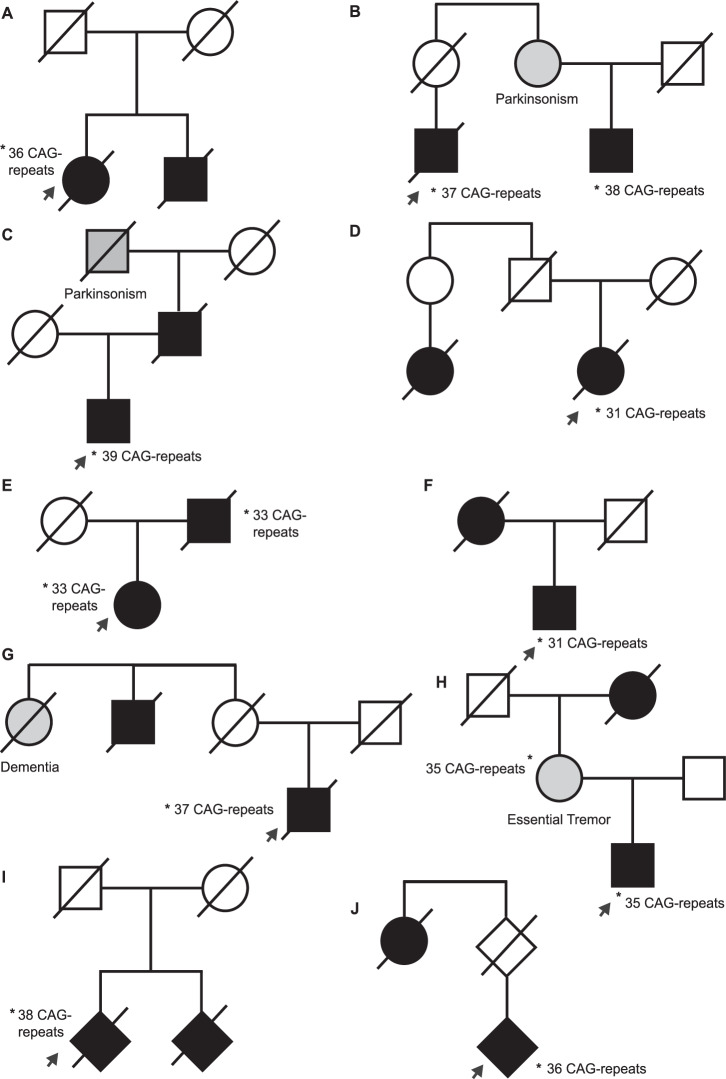
Pedigrees involving familial ALS and CAG-repeat expansion of *ATXN2.* In each pedigree the arrow indicates the index case. Black shading indicates individuals with a clinical diagnosis of ALS; grey shading indicates a diagnosis of another neurodegenerative phenotype as indicated in the accompanying text. *indicates individuals with DNA available and a confirmed genetic diagnosis of a full-length CAG repeat expansion of *ATXN2;* unfortunately testing was not possible in unaffected relatives. A diamond is used where biological sex was not available. **A** Utrecht A pedigree; **B** Utrecht B pedigree; **C** Utrecht C pedigree; **D** Utrecht D pedigree; **E** Utrecht E pedigree; **F** Utrecht F pedigree; **G** Montreal pedigree; and **H** Sheffield pedigree; **I, J** PreventionGenetics pedigrees.

This was a White European female in her 70s, with a past medical history of type 2 diabetes mellitus. She presented with bulbar weakness and later she developed limb weakness. No cerebellar symptoms were identified. She was diagnosed with clinically definite ALS according to revised El-Escorial criteria, and she eventually died from respiratory failure 9.4 years after symptom onset. Her brother also had ALS and died aged in his 80s (Fig. 1A). Genotyping of the index case revealed a heterozygous 36 CAG-repeat expansion in *ATXN2*.

#### Utrecht B

This pedigree (Fig. 1B) included a White European male in his 60s, with a past medical history of atrial fibrillation, obstructive sleep apnea and primary hyperparathyroidism. He presented with upper limb weakness leading to a diagnosis of clinically probable ALS. Detailed inspection revealed no evidence of cerebellar atrophy on brain MRI. He had rapidly progressive disease and died from respiratory failure 11.5 months after symptom onset. His maternal cousin suffered bulbar-onset ALS in his 60s, five years after he had been diagnosed with parkinsonism. He is still alive more than 3 years after the onset of ALS symptoms. The mother of the index case died from cancer before the age of sixty. The aunt of the index case (the mother of the patient with parkinsonism) (Fig. 1B) also had a diagnosis of parkinsonism with onset in her 60s but no DNA was available for genotyping. The index case carried a heterozygous 37 CAG-repeat *ATXN2* expansion including two CAA interruptions: NM_002973.4:c.16CAG[14]CAA[1]CAG[13]CAA[1]CAG[8], and his cousin carried a 38 CAG-repeat expansion but we were unable to confirm the presence of CAA interruptions.

#### Utrecht C

This was a White European male in his 70s who presented with spastic gait and pseudobulbar affect with evidence of weakness and fasciculations in all four limbs, leading to an El-Escorial classification of probable ALS, laboratory supported. MRI of the brain and spinal cord did not reveal an alternative cause of his symptoms and of note, no cerebellar atrophy. The patient is alive more than three years after symptom onset. The father of the index case suffered similar symptoms in his 60s and died 12 years after symptom onset, presumably from ALS although we could not confirm this diagnosis; the paternal grandmother of the index case died with a diagnosis of parkinsonism (Fig. 1C). Genotyping of the index case revealed a heterozygous 39 CAG-repeat expansion of *ATXN2* including four CAA interruptions: NM_002973.4:c.16CAG[8]CAA[1]CAG[9]CAA[1]CAG[4]CAA[1]CAG[4]CAA[1]CAG[10].

### Montreal pedigree

This was a French-Canadian male in his 60s, born from consanguineous parents, who presented with slow walking and instability with falling. He was subsequently diagnosed with typical ALS. The patient had an uncle who also suffered from ALS and an aunt who was diagnosed with Alzheimer’s dementia (Fig. 1G). Genotyping of the index case revealed a heterozygous 37 CAG-repeat expansion of *ATXN2*. Survival was relatively brief at only 17 months from symptom onset.

### Sheffield pedigree

A White European male in his 30s presented with a two-month history of left leg weakness. Examination revealed weakness and spasticity with pathologically brisk reflexes in both lower limbs. There was no evidence of ataxia or abnormal eye movements. MRI brain scans showed high T2 signal intensity along both corticospinal tracts and detailed inspection revealed no evidence of cerebellar atrophy. A diagnosis of clinically probable ALS was made according to revised El-Escorial criteria.

The patient’s family history (Fig. 1H) was significant for ALS through his maternal grandmother. She developed bulbar onset ALS in her mid-sixties and died due to respiratory failure after two years. The patient’s mother is a relatively well female in her 70s, in whom neurological examination revealed only signs that were suggestive of essential tremor (ET). We characterised the inheritance pattern as autosomal dominant with variable penetrance. Genetic testing revealed an *ATXN2* expansion of 35 CAG-repeats in one allele; and a normal length 23 CAG-repeat in the other allele, in both the proband and his mother. The expanded allele contained five CAA interruptions in both the proband and his mother: NM_002973.4:c.16CAG[8]CAA[1]CAG[4]CAA[1]CAG[4]CAA[1]CAG[4]CAA[1]CAG[4]CAA[1]CAG[6].

### Intermediate length expansions

#### Utrecht D

This was a White European female in her 70s who presented with respiratory failure, weakness in all four limbs and dysphagia. She was subsequently diagnosed with mixed site of onset ALS. She met El-Escorial criteria for possible ALS. Family history of ALS was notable for an affected cousin (Fig. 1D). The index case died due to respiratory failure 6.7 years after symptom onset. Genotyping in DNA available from the index case revealed a heterozygous 31 CAG-repeat expansion of *ATXN2*.

#### Utrecht E

This was a White European female in her 50s who presented with weakness and fasciculations in both legs. She was diagnosed with the progressive muscular atrophy (PMA) variant of ALS 4.5 years after symptom onset. Her father was also diagnosed with the PMA variant ALS and died in his 80s, 6.5 years after symptom onset (Fig. 1E). Genotyping of both cases revealed a heterozygous 33 CAG-repeat expansion of *ATXN2*; the index case carried a single CAA interruption: NM_002973.4:c.16CAG[23]CAA[1]CAG[9]; but we could not confirm this in her father.

#### Utrecht F

This was a White European male who presented in his 60s with weakness, atrophy and fasciculations in both legs and his left arm. He was diagnosed with clinically definite ALS nine months after a symptom onset. His mother was also diagnosed with ALS in her 50s and survived three years (Fig. 1F). Genotyping in DNA available from the index case revealed a heterozygous 31 CAG-repeat expansion of *ATXN2*.

**Sporadic ALS** cases associated with full-length *ATXN2* expansions are detailed in Table 1. In Cases 1-5 (Table 1) we were able to confirm the absence of cerebellar atrophy via the independent assessment of two neuroradiologists blinded to the diagnosis. In two cases (Table 1, Patient Number 3 and Patient Number 9) we confirmed the presence of a 37 CAG-repeat expansion of *ATXN2* with four CAA interruptions.

**Table 1:** Sporadic ALS presentations associated with full-length CAG-repeat expansions of *ATXN2*.

Patient Number	Sex	ATXN2 genotype	Interruptions expanded allele (position)	Extramotor symptoms	Site of Onset	Age of Onset	Survival	Cerebellar atrophy	Family history
1	Male	22/39	NA	None	Spinal	70 s	3	No	None
2	Male	22/38	NA	None	Spinal	70 s	0.6	No	None
3	Male	22/37	9,19,24,29	None	Spinal	60 s	2.5	No	Dementia
4	Female	22/37	NA	None	Spinal	50 s	>2	No	Dementia
5	Female	22/35	NA	None	Spinal	80 s	0.7	No	None
6	Male	22/34	9	None	Spinal	60 s	4.3	NA	Dementia
7	Male	22/36	28	None	Bulbar	60 s	>1.5	NA	None
8	Male	22/36	None	None	Spinal	60 s	>1	NA	None
9	Male	22/37	9,14,19,29	None	Spinal	40 s	3.3	NA	None
10	Female	22/34	9	None	Spinal	30 s	6.1	NA	None
11	Male	22/35	9	None	Spinal	60 s	2.5	NA	None
12	Female	27/34	9	None	Spinal	60 s	4.4	NA	None
13	Female	22/34	None	None	Spinal	70 s	3.2	NA	None
14	Male	22/37	None	None	Bulbar	60 s	>2.5	NA	None
15	Male	22/38	9	None	Spinal	70 s	NA	NA	None

Additional ALS-associated pathogenic variants were excluded in *C9ORF72* for all patients. For Patient Number 3 disease-associated mutations were also excluded within *ANXA11, FUS, GRN, KIF5A, NEK1, TBK1, OPTN, PFN1, SOD1, TARDBP, TUBA4A, UBQLN2* and *VCP*. Cerebellar atrophy was assessed by two independent neuroradiologists who were blinded to the diagnosis.

### Cohort study

**Fig. 2:**
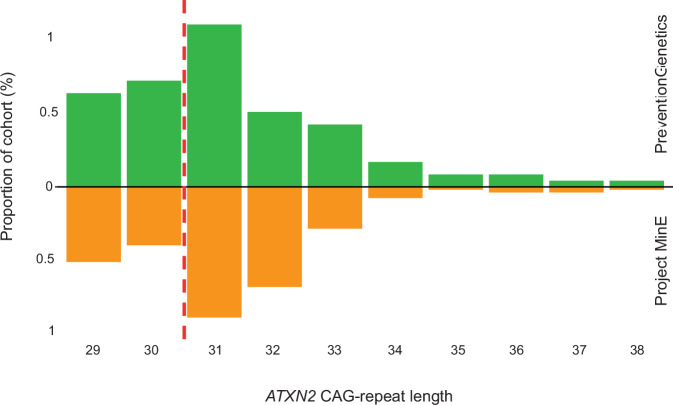
Cohort study of *ATXN2* CAG-repeat frequency in ALS patients. We present results from genotyping in 2322 patients who presented with ALS from PreventionGenetics (green) and 5242 patients diagnosed with ALS, primary lateral sclerosis (PLS), progressive muscular atrophy (PMA), progressive bulbar palsy (PBP) or ALS-FTD from project MinE (orange). The graph illustrates the proportion of intermediate and full length CAG-repeat expansions in a representative ALS population. The red line signifies the cut-off between traditional designations of normal and intermediate length expansions.

We also present summary data from a cohort of patients who presented with ALS, who have been subjected to comprehensive ALS genetic testing at a commercial clinical laboratory (PreventionGenetics, a part of Exact Sciences) including the set of ALS genes listed in Supplementary Table 1. Specifically, 57 of 2,262 (2.5%) samples that were processed for ALS testing carried an *ATXN2* repeat length of ≥31 (Fig. 2). Of these 57, ten (0.4%) patients harboured *ATXN2* repeat length of ≥34. Age of onset for these patients was in the typical range. Of the ten patients, six did not mention whether they had any family history, two explicitly stated that there was no known family history, and two reported a family history of ALS. Of these two, one patient carried a single expansion of *ATXN2* containing 38 CAG-repeats and had a sibling with ALS (Fig. 1I), although details of genetic testing in the sibling were not available. The other patient featured an *ATXN2* CAG-repeat expansion of length 36 and reported to have an aunt who suffered ALS (Fig. 1J), but no other information regarding genetic testing in the aunt was provided. No MRI brain imaging data provided, but all patients tested negative for ALS-associated genetic changes within *C9ORF72, SOD1, FUS*, and *TARDBP*. Any patients specifically tested for a clinical diagnosis of spinocerebellar ataxia were not included in this analysis in order to provide prevalence estimates for a typical ALS cohort. Information on CAG-repeat interruptions was not available from this cohort.

We verified the frequencies of *ATXN2* expansions via testing in the Project MinE cohort (www.projectmine.com) [11] consisting of 5242 patients diagnosed with ALS, primary lateral sclerosis (PLS), progressive muscular atrophy (PMA), progressive bulbar palsy (PBP) or ALS-FTD. Similar to our primary cohort analysis, 108 patients carried an *ATXN2* repeat length of ≥31 (2.1%), of which 10 (0.2%) harboured *ATXN2* repeat length of ≥34 (Individuals 6-15 in Table 1) (Fig. 2). Six of the 10 patients carried a single CAA interruption within their expanded *ATXN2* allele, and one patient with a CAG-repeat length of 37 had four separate CAA interruptions. Importantly three of the 10 patients had no interruptions within their expanded *ATXN2* allele and all three of these patients had a diagnosis of ALS. We note that Project MinE participants are selected for sporadic rather than familial disease [11], which may have reduced the representation of full-length *ATXN2* expansions compared to the PreventionGenetics cohort.

As a comparison we genotyped *n* = 2425 controls from the Project MinE cohort who are matched by population and genotyping method to the Project MinE ALS patients. Nine controls carried an *ATXN2* repeat length of ≥31 (0.4%), of which 1 (0.04%) harboured *ATXN2* repeat length of 34 or greater. This control carried a single expanded *ATXN2* allele with 37 CAG-repeats and was last known to be healthy more than ten years ago, aged in her 50s; this is beyond the typical age of onset for SCA2 but is approaching the peak age of onset for ALS. The control frequency we report is similar to that reported in a recent meta-analysis where the equivalent figures were: 1.3% and 0.08% [6].

The difference in frequency of *ATXN2* expansions between cases and controls was significant for all *ATXN2* expansions of CAG-repeat length of ≥31 (PreventionGenetics: OR = 6.9, *p* = 1.0e-10, Fisher’s exact test; Project MinE: OR = 5.6, *p* = 6.0e-10); and for full-length expansions of 34 or greater (PreventionGenetics: OR = 10.5, *p* = 5.3e-3; Project MinE: OR = 4.6, *p* = 0.2).

## Materials and methods

### *ATXN2* genotyping

Genotyping was performed by exome sequencing except in Project MinE where whole genome sequencing was employed as described previously [11]. For exome sequencing: Exons were captured including ~10 bases of non-coding DNA flanking each exon. Captured DNA is sequenced on the NovaSeq 6000 using 2 × 150 bp paired-end reads (Illumina, San Diego, CA, USA). >97% of target bases were covered at >20x, and the mean coverage of target bases >100x. Variant calls are made by the GATK Haplotype caller. *ATXN2* CAG-repeat length was measured with ExpansionHunter, version 5.0.0 [12], and the motif interruptions were analysed with REViewer [13]. The exception was the Sheffield pedigree where sizing of the CAG‐repeat in *ATXN2* was performed by in-house fluorescent PCR followed by sizing and analysis on an ABI 3730 DNA Analyzer with Genemapper software v4.0 (Applied Biosystems).

## Discussion

The identification of *ATXN2* expansions associated with both familial and sporadic ALS, irrespective of CAG-repeat length, challenges current notions. This suggests that the traditional understanding which links intermediate-length *ATXN2* expansions to sporadic ALS, and full-length expansions to SCA2, is not an absolute rule, even though it applies to the majority of cases. Cases of SCA2 associated with intermediate length *ATXN2* expansions have been described [5, 14, 15]. Here we describe a series of both familial and sporadic ALS patients with either intermediate or full-length *ATXN2* expansions. Our cohort analyses demonstrate that both intermediate and full-length *ATXN2* expansions are present at a greater than five-fold higher rate in ALS patients compared to controls. We also showed that multiple *ATXN2*-associated phenotypes can be present within the same pedigree or even the same patient, including ALS, parkinsonism and perhaps even ET, although it is difficult to exclude the possibility that the latter was a mild form of SCA2 presenting with subtle ataxia. We conclude that SCA2, ALS and parkinsonism associated with CAG-repeat expansion in *ATXN2* should be considered an example of pleiotropy and not distinct entities based on CAG-expansion length. A better framing might be to refer to ‘*ATXN2*-related neurodegeneration’, to capture the potential clinical manifestations of CAG-repeat expansion of *ATXN2* within a patient or pedigree.

Interrupted repeats have been associated with ALS and parkinsonism [16, 17] rather than with the SCA2 phenotype. Unfortunately we were not able to assess the presence of CAG-repeat interruptions in all patients. However, ten ALS patients, of whom three had a confirmed family history of ALS, carried CAA interruptions within the *ATXN2* CAG-repeat expansion. In contrast, we also identified three ALS patients without CAA interruptions. Moreover, there are a number of reports of SCA2 and ALS associated within a single pedigree/genotype [7, 8, 18]; in these cases it is assumed that the same *ATXN2* genotype, whether or not interruptions are present, has led to both ALS and SCA2. We conclude that CAG-repeat interruptions are frequently associated with an ALS phenotype, but there is not a simple dichotomy and that ALS can be associated with either interrupted or uninterrupted expansion of *ATXN2*. The key to unravelling these questions will be a molecular understanding of the role of *ATXN2* expansions. We note recent work [19] suggesting that repeat expansions of *ATXN2* are sufficient to induce mitochondrial dysfunction and induce disease-associated microglial activation. It would be interesting to understand the relative effect of interrupted and uninterrupted expansions on these phenotypes.

Another question refers to penetrance. Here, we report one full-length (Fig. 1C) and two intermediate-length *ATXN2* expansion pedigrees (Fig. 1E, F) that are suggestive of autosomal dominant inheritance including male-to-male transmission (Fig. 1C). Additional genetic and environmental characterisation of our patients was limited and it is impossible to be sure what other additional risk factors, whether genetic or environmental, may have influenced penetrance of the *ATXN2* expansion in the patients we describe. However, we have excluded other highly-penetrant genetic causes of ALS in two of the three pedigrees consistent with autosomal dominant inheritance (Supplementary Table 1). Our interpretation is that *ATXN2* CAG-repeat expansions, irrespective of length, should be considered a risk factor for both familial and sporadic ALS in a similar manner to G4C2-repeat expansion within *C9ORF72* [10] although not with the same effect-size.

Observed anticipation of phenotypes linked to repeat expansions is good evidence for a link between repeat length and penetrance/phenotype. For SCA2, there is evidence of anticipation of approximately 14 years per generation [20]. We do not find evidence for anticipation in the cases we report; only in two pedigrees (Fig. 1E, H) was the age of onset earlier in the index case compared to prior generations, and even here we have information from a limited number of individuals. We conclude that penetrance, and age of onset, are determined by multiple factors of which CAG-repeat length may be a contributor, but further work is required to determine which other genetic and environmental factors are important.

A limitation of our study is incomplete clinical documentation of assessment of ataxia in some of the patients diagnosed with ALS. Motor neuron degeneration is associated with the SCA2 phenotype [21] and it is conceivable that ALS may be mistakenly undiagnosed in a patient already suffering from SCA2. We have attempted to circumvent this possibility in a subset of patients where we could provide neuroimaging evidence for an absence of cerebellar degeneration at the time of ALS diagnosis, but this was not possible for all cases. Finally, we mostly utilised Illumina short-read sequencing together with ExpansionHunter analysis to size the *ATXN2* CAG-repeat. Although imperfect this method has reasonable accuracy [22], but this could conceivably have affected the identification of *ATXN2* expansions in borderline cases.

**Fig. 3:**
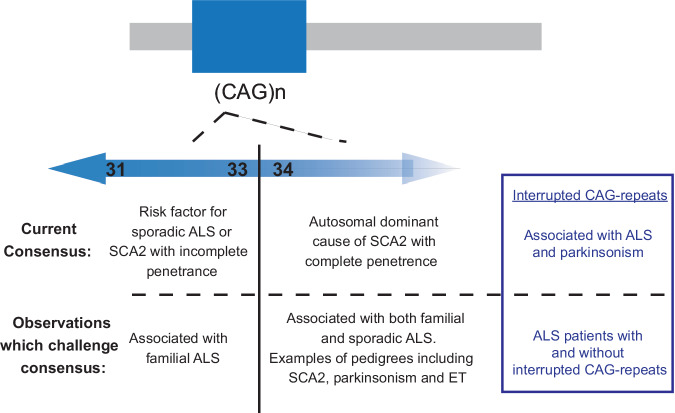
Key changes proposed to the current consensus regarding phenotypes associated with the CAG-repeat expansion of *ATXN2* genotype. Previous consensus has been that 31–33 CAG-repeats is associated with sporadic ALS and a full length expansion of ≥34 CAG-repeats is associated with SCA2 with complete penetrance. While this is likely to be true in the majority of cases we have observed ALS associated with both intermediate and full-length *ATXN2* CAG-repeat expansions, including where there is a positive family history to suggest high penetrance. Pedigrees include additional neurodegenerative phenotypes suggesting pleiotropy, and ALS is not exclusively associated with interrupted CAG-repeats.

Our conclusions regarding pleiotropy and penetrance are summarised in Fig. 3. In our cohort study we show that the associations we describe between ALS and intermediate or full-length *ATXN2* expansions, are not infrequent and are thus relevant for clinical practice. Our work has significant implications for genetic counselling of families affected by *ATXN2*-related neurodegeneration, although we did not have sufficient numbers to assess the relative risk of each phenotype for a given expansion length and composition.

## Supplementary information

Supplementary Table 1

## Data Availability

Sequencing data are available upon request. The Project MinE WGS data are accessible upon application approval (https://www.projectmine.com/research/data-sharing/). Genotype frequency information are publicly available at http://databrowser.projectmine.com/.
